# A Biphasic Pleural Tumor with Features of an Epithelioid and Small Cell Mesothelioma: Morphologic and Molecular Findings

**DOI:** 10.1155/2016/1532424

**Published:** 2016-06-15

**Authors:** Sarah Hackman, Richard D. Hammer, Lester Layfield

**Affiliations:** Department of Pathology and Anatomical Sciences, University of Missouri, M263 Medical Science Building, One Hospital Drive, Columbia, MO 65212, USA

## Abstract

Malignant mesotheliomas are generally classified into epithelioid, sarcomatoid, desmoplastic, and biphasic types with rare reports of a small cell form. These small cell variants display some morphologic overlap with desmoplastic small round cell tumors (DSRCTs) which generally occur within the abdominal cavity of young males and are defined by a characteristic t(11;22)(p13;q12) translocation. However, there are rare reports of DSRCTs lacking this translocation. We present a 78-year-old man with a pleura-based biphasic neoplasm with features of both epithelioid mesothelioma and a small cell blastema-like neoplasm. The epithelioid portion showed IHC reactivity for pan cytokeratin, CK5/6, D2-40, and calretinin and the small cell portion marked with CD99, pan cytokeratin, WT1, FLI1, S100, CD200, MyoD1, and CD15. Fluorescence in situ hybridization testing for the t(11;22)(p13;q12) translocation disclosed loss of the* EWSR1* gene in 94% of tumor cell nuclei, but there was no evidence of the classic translocation. Array based-comparative genomic hybridization (a-CGH) confirmed the tumor had numerous chromosome copy number losses, including 11p15.5-p11.12 and 22q12.1-q13.33, with loss of the* EWSR1* and* WT1* gene regions. Herein, we report novel complex CGH findings in a biphasic tumor and review the molecular genetic alterations in both mesothelioma and DSRCTs.

## 1. Introduction

Primary tumors of the pleura are relatively uncommon and are divided by the World Health Organization (WHO) 2014 classification into mesothelial tumors, lymphoproliferative disorders, and mesenchymal tumors [1, 2]. Primary neoplasms with a small cell morphology arising within the pleura are rare and include desmoplastic small round cell tumors (DSRCTs) [2, 3] and pleuropulmonary blastomas [4]. The existence of a true small cell variant of malignant mesothelioma is controversial with only rare case reports and two small series being reported [5–7]. The small cell type of mesothelioma was in the 2004 WHO classification [8, 9] but is not mentioned in the 2014 WHO classification [1]. While pleuropulmonary blastomas occur almost exclusively in children [4], DSRCTs can occur in the pleura of adults and may be confused with mesotheliomas including the small cell type [9]. Desmoplastic small round cell tumors (DSRCTs) were first described by Gerald et al. in 1991 as an unusual, highly malignant neoplasm occurring within the abdominal cavity of young males predominantly [10]. Subsequent authors confirmed the histologic appearance of small round cells arranged in nests and sheets surrounded by a desmoplastic stroma [11]. Additional studies have extended both the age range and sites of origin to include pleural serosa, paratesticular region, ovary, posterior cranial fossa, and other soft tissues and bone [12, 13]. DSRCTs demonstrate simultaneous coexpression of epithelial, neural, and muscle immunohistochemical (IHC) markers. The tumors are commonly immunoreactive for cytokeratin, epithelial membrane antigen (EMA), vimentin, and neuron-specific enolase (NSE) and have a punctate perinuclear dot-like Golgi pattern positivity for desmin [13–16]. Most DSRCTs also show reactivity with WT1, FLI1, and CD99 [13, 15, 17]. Occasionally, there can be phenotypic overlap or atypical immunohistochemical expression that may mimic other small round cell tumors or malignant mesothelioma [13].

Cytogenetic and molecular studies are often required to accurately distinguish DSRCT from other small blue cell neoplasms. Approximately, 96% of DSRCTs have a characteristic chromosomal translocation t(11;22)(p13;q12) that produces a fusion of* EWS* and* WT1* genes [18]. There have been rare reports of DSRCTs with variant translocations or lacking of the translocation [19–21].

Here we describe a patient's tumor with histopathological and immunohistochemical features of a mixture of a small cell malignancy and an epithelioid mesothelioma that had multiple complex chromosomal abnormalities on microarray, including the loss of 11p15.5-p11.12 and 22q12.1-q13.33 regions. These deletions, involving the* EWS* and* WT1* genes, were described in one of the first karyotyped cases of DSRCTs in 1993 and 1994 [19, 20]. Based on the mixed morphology of a small cell malignancy and an intimately associated papillary patterned mesothelioma composed of cells lacking the t(11,22) translocation, we believe our own case represents a small cell variant of mesothelioma. The relationship between small cell mesothelioma and DSRCTs is unclear.

## 2. Case Presentation

### 2.1. Clinicopathological Findings

A 78-year-old Caucasian man presented to the emergency department with a two-week history of shortness of breath, nonproductive cough, decreased exercise tolerance, and fatigue. His past medical and surgical history was significant for gastrointestinal reflux disease and resection of a stage I melanoma from his scalp. He had no exposure to cigarette smoke or asbestos. Physical examination revealed decreased breath sounds and dullness to percussion over his right hemithorax. Laboratory values were within normal limits except for an elevated creatinine. Chest X-rays disclosed a large right-sided pleural effusion with right middle and lower lobe collapse. There was a 4.4 × 3.0 × 4.2 centimeter (cm) pleura-based, enhancing lesion adjacent to the collapsed right lung and a second 1.6 × 1.0 × 1.2 cm pleura-based lesion. The patient was admitted to the hospital and underwent multiple thoracentesis procedures to drain 4.2 liters of fluid. Samples were sent to cytology for analysis.

**Figure 1:**
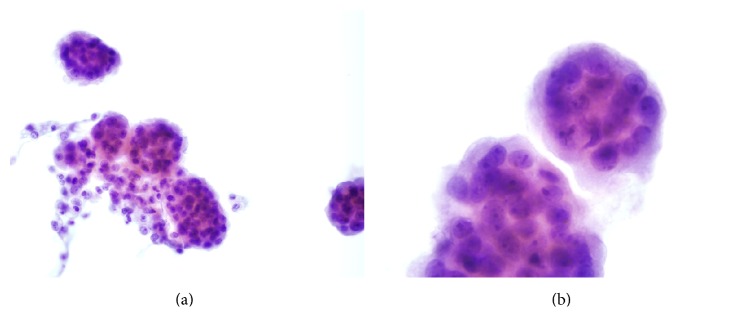
(a) Pleural fluid containing multiple spheres of neoplastic cells, Papanicolaou ×400. (b) Cell ball composed of tightly packed atypical oval cells with irregular hyperchromatic nuclei, Papanicolaou ×1000.

Review of cytospin preparations of the pleural fluid revealed numerous well-formed spheres of atypical cells. The spheres were composed of relatively large oval to polygonal cells with moderate amounts of cytoplasm surrounding large nuclei with partial chromatic clearing and distinct nucleoli (Figures 1(a) and 1(b)). A diagnosis of “highly suspicious for malignant mesothelioma” was made.

**Figure 2:**
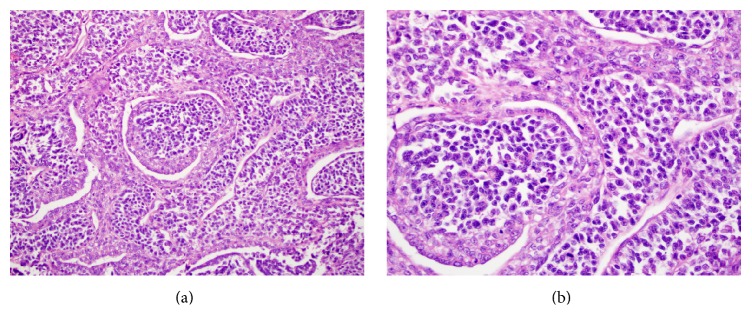
(a) Biphasic tumor with papillary fronds lined by large atypical mesothelial cells, ×200. (b) Neoplastic mesothelial cells with moderate amount of cytoplasm and large nuclei showing partial chromatic clearing and distinct nucleoli, ×600.

**Figure 3:**
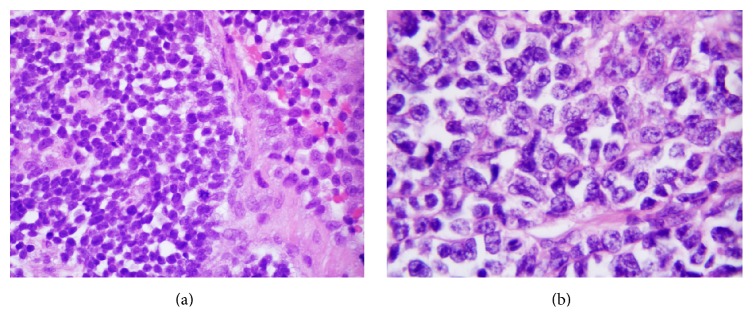
(a) The small round cell population arranged in nests of cells with scant cytoplasm and slightly irregular nuclei, ×600. (b) Small cell component with scant cytoplasm and hyperchromatic nuclei containing distinct nucleoli, ×1000.

**Figure 4:**
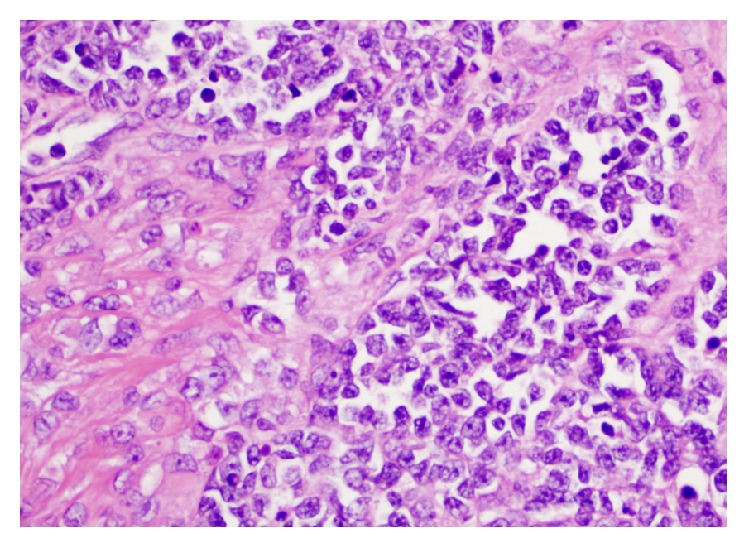
The small cell component was occasionally associated with a sclerotic stroma, ×400.

**Figure 5:**
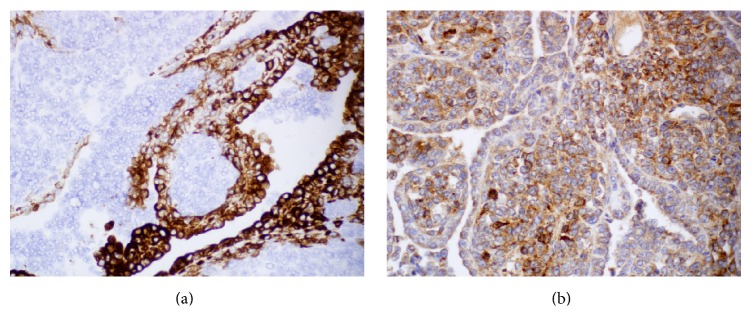
(a) Surface mesothelial cell component strongly reactive for calretinin, immunohistochemistry ×400. (b) Both the small cell component and the larger lining cells were reactive with antibodies against CD99, immunohistochemistry ×400.

**Figure 6:**
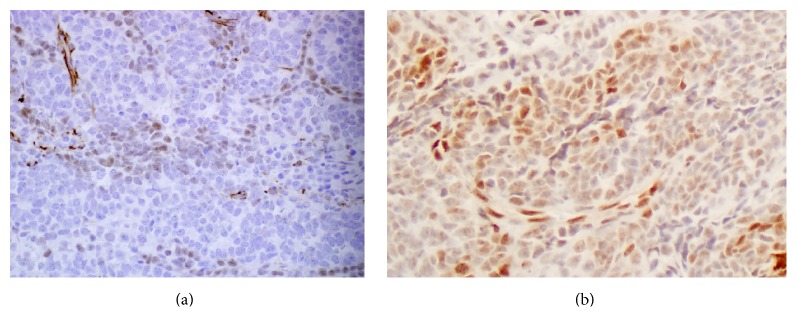
(a) Small cell component weakly and focally reactive for WT1, immunohistochemistry ×400. (b) Antibodies against FLI1 decorated a component of the small cell population, immunohistochemistry ×200.

A right video-assisted thoracoscopic surgery was performed and the mass biopsied. Histologic evaluation of the biopsy specimen revealed a biphasic tumor characterized by papillary structures and cell nests composed of large polygonal cells with moderate amounts of cytoplasm surrounding large nuclei. The nuclei had a granular to cleared chromatin and distinct nucleoli (Figures 2(a) and 2(b)). The majority of the neoplasm was composed of a small round cell population (Figures 3(a) and 3(b)). The small round cells had scant cytoplasm and hyperchromatic nuclei. These small round cells were arranged in sheets and nests. Some nests were surrounded by a sclerotic stroma (Figure 4). Immunohistochemical staining demonstrated the larger cells to be CK5/6, pan cytokeratin, calretinin, D2-40, and CD99 positive (Figures 5(a) and 5(b)), while the small cell component was immunoreactive for CD99, WT1, FLI1, CD15, cytokeratin, cytoplasmic Golgi MyoD1, and focally S100 (Figures 6(a) and 6(b)). Both cell populations were nonreactive for desmin and numerous other immunostains (Table 1).

A PET/CT examination was performed which confirmed the presence of multiple right sided pleura-based masses, but there was no metastatic disease. The patient agreed to begin Carboplatin/Pemetrexed chemotherapy.

### 2.2. Molecular Findings

**Figure 7:**
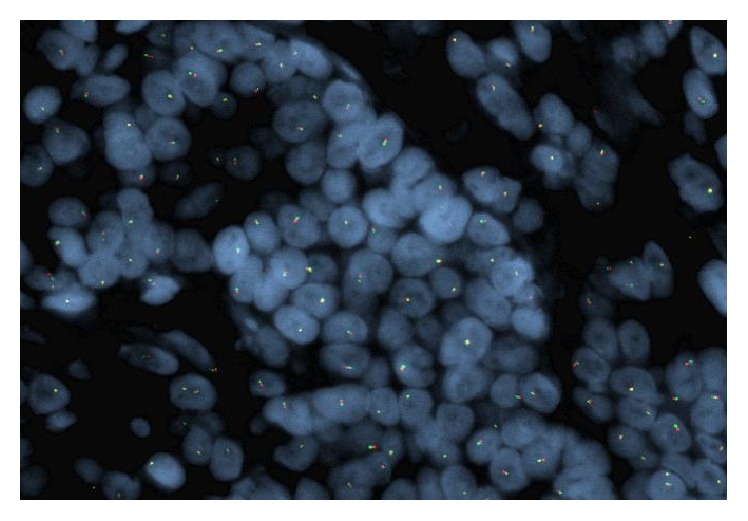
Dual color break-apart FISH probe with only one intact EWSR1 signal in majority of nuclei.

Fluorescence in situ hybridization (FISH) testing was performed on 100 interphase cells from the pleura-based tumor using dual color break-apart probes for 5′*EWSR1* and 3′*EWSR1* gene regions at 22q12. 94% of nuclei had only a single intact copy of the EWSR1 fusion signal, but 0% of nuclei had separation of the 5′ and 3′ signals. Although no rearrangement of the* EWSR1* gene region was seen, the widespread loss of signal was suspicious for complex chromosomal alterations involving either one or both of the translocation sites (Figure 7). An array of comparative genome hybridization (a-CGH) was performed on formalin-fixed paraffin embedded (FFPE) slides to investigate the possibility of monosomy 22 or deletion of the* EWSR1* gene region.

The a-CGH revealed numerous abnormalities. In addition to the loss of heterozygosity (LOH) of 6p and 6q, there were deletions and pathogenic losses on chromosomes 1, 3, 5, 6, 9, 10, 11, 13, 17, 18, 20, and 22 and the distal long arm of the Y chromosome.  Table 2 describes the genomic coordinates (hg19), the sizes, and the genes involved. The losses of chromosome regions 11p13 and 22q12 implies a loss of the* WT1* and* EWSR1* genes, respectively, and confirms that the single intact signal seen on FISH was caused by deletion of the* EWSR1* gene region.

Reverse transcriptase polymerase chain reaction (RT-PCR) was performed at an outside reference laboratory using primers specific for the* EWSR1-WT1*,* EWSR1-FLI1*, and* EWSR1-ERG* fusion transcripts after the slide was macrodissected for tumor enrichment. PCR analysis with the appropriate positive, negative, and blank controls revealed that no fusion transcripts were present.

## 3. Discussion

Morphologically, our case presented a mixed pattern composed of both a papillary pattern epithelioid mesothelioma and a larger small round cell component. This small cell component raised the possibility of a DSRCT or a small cell predominant mesothelioma as described by Mayall and Gibbs [5], Ordóñez [6], and Cha et al. [7].

The majority of prior reports of small cell mesothelioma describe a micropapillary or tubulopapillary component composed of cells with eosinophilic cytoplasm [6, 7]. This papillary component often invaded the surrounding tissue and could demonstrate lymphovascular invasion [7]. In our case, the non-small cell component formed papillary structures composed of large polygonal cells with granular or clear cytoplasm similar to that described by Ordóñez [6]. While the majority of cases described by Ordóñez [6] had a low mitotic index (<5 mitoses per 10 high-power fields), our case and that reported by Cha et al. [7] had frequent mitotic figures. In reported cases, the proportion of the small cell component varied from 80 to 100% in biopsy material to 15 to 20% in resection specimens. In our case, the majority of the tumor was composed of the small cell component. In one case reported by Ordóñez [6], the small cell component was composed of cell nests separated by a myxoid matrix. Our case had similar myxoid areas as well as zones of stromal sclerosis suggesting the pattern seen in some DSRCTs. Histologically, DSRCTs are classically composed of discrete nests of small blue cells with intervening desmoplastic stroma [12, 18]. The amount of desmoplastic stroma is variable: tumor cells can be in trabeculae or single file if the sclerotic stroma is abundant or can appear as diffuse sheets if the desmoplastic stroma is scarce or nearly absent [13, 14]. Uncommonly, tubules, glands, and rosette-like structures have also been observed [13, 18]. Cytologically, DSRCTs are composed of medium-sized cells with hyperchromatic nuclei and inconspicuous nucleoli [12, 15]. Because DSRCTs have been reported in a variety of anatomic sites and the histological features of this tumor may overlap with other small blue cell tumors, immunohistochemistry is often the first tool used to narrow the differential diagnoses.

Although exact percentages of immunopositivity vary, DSRCTs are routinely positive for cytokeratin, EMA, NSE, vimentin, and desmin. EMA positivity is seen in greater than 90% of DSRCTs and is the marker of choice for epithelial differentiation [14]. Although desmin reactivity is characteristically perinuclear and dot-like, there have been cases where it is not prominent [12]. Up to 19% of tumors have been reported to lack desmin positivity as in our case [18]. A study by Lae et al. reported that 91% of DSRCTs were positive for WT1 [18]. Other markers are less commonly seen. One study of 23 DSRCTs reported 57% reactivity for CD99, 81% reactivity for placental alkaline phosphatase, 29% reactivity for myogenin, 0% reactivity with MyoD1, and up to 19% with calretinin [22].

The immunostaining pattern for mesotheliomas is considerably more straightforward than the complex epithelial, neural, and muscle coexpression in DSRCTs. Although the majority of both DSRCTs and mesotheliomas express WT1, only rarely and weakly do DSRCTs express calretinin [14]. Some have hypothesized that calretinin positivity in DSRCTs may be related to focal mesothelial differentiation within a DSRCT or enveloping of mesothelial cells by a surrounding DSRCT [14]. Additionally, mesotheliomas generally show immunopositivity for CK5/6, CK7, and HBME-1 and are negative for desmin [23, 24]. Rare cases of mesothelioma with small cell morphology have been described and show some morphological and immunophenotypic overlap with our patient's tumor [5, 6]. As seen in Table 1, our patient's tumor showed a biphasic staining pattern and the larger cells had a mesotheliomatous immunoprofile with positivity for calretinin, CK7, CK5/6, and WT1 which was definitively distinct from the smaller blue cell component.

Immunohistochemical findings in the reported small cell mesothelioma have been variable, but Cha et al. [7] report that the micropapillary component demonstrated weak staining for calretinin and the small cell component being entirely negative. TTF-1 was entirely negative with the tumor. WT1 demonstrated strong and defuse nuclear staining in both the papillary and small cell components. Ordóñez [6] demonstrated similar findings. In our case, the papillary or large cell population showed a staining pattern characteristic for mesothelioma with calretinin, cytokeratin, and CD99 positivity, while the small cell component was positive for CD99, WT1, FLI-1, and cytokeratin. The positivity for pan cytokeratin and MyoD1 raised the possibility that the small cell component represented differentiation to DSRCT. WT1, and FLI-1, and CD99 positivity also suggested the possibility of differentiation to DSRCT.

There have been several reports of aberrant or unusual DSRCT immunostaining [16, 25, 26]. One reported case with minimal desmoplasia was negative for WT1 and CD99 and had only 5% staining with EMA and patchy weak desmin staining with “scanty” perinuclear dot-like positivity [26]. However, FISH studies confirmed EWS-WT1 gene fusion and the diagnosis of DSRCT in that case. As seen in Table 1, our patient's tumor did not show desmin staining but was immunopositive for MyoD1. Interestingly, MyoD1 mimicked the often described desmin-type pattern of reactivity with punctate Golgi positivity in the small blue cell areas. MyoD1 is an early marker of muscle differentiation and may be acting as a surrogate for desmin positivity in our case. The variability in immunostaining patterns among these tumors makes molecular studies in cases of suspected DSRCT paramount for correct classification [26].

Unlike prior studies of small cell mesotheliomas, we were able to perform a number of molecular analyses in our case. These studies were performed to determine whether the small cell component demonstrated features consistent with a primary mesothelioma or a DSRCT. Molecular studies, including FISH or RT-PCR, are designed to detect the recurrent t(11;22)(p13;q12) translocation that generates a* EWS-WT1* fusion protein [21, 27]. The protein is formed when the N-terminal of the* EWS* gene combines with the C-terminal, DNA-binding domain of* WT1*. This fusion most commonly involves* EWS* exons 1–7 and* WT1* exons 8–10.* WT1* normally functions as a zinc-finger transcription factor that acts as a tumor suppressor. If the fusion product is formed, the transcription suppression of* WT1* is lost and the fusion gene becomes an aberrant transcription factor [27, 28]. Gerald and Haber reviewed several downstream targets affected by the EWS-WT1 fusion [29]. Platelet derived growth factor A chain (PDGFA) is induced by the fusion product and acts as a weak oncogene and a chemoattractant for fibroblasts [29]. This genetic interaction may explain the desmoplasia seen histologically in DSRCTs [29]. Interleukin 2 receptor (IL-2R), myelodysplasia/myeloid leukemia factor 1 gene (MLF1), and T-cell acute lymphoblastic leukemia-associated antigen 1 (TALLA-1) are thought to be other downstream targets of the fusion product which may contribute to cell growth, resistance to apoptosis, and migration and invasion, respectively [29].

Molecular variants of the* EWS-WT1* fusion have been reported in up to 5% of DSRCTs, but the clinicopathological significance of these variants is not known [18, 27]. The most common type of variation is additional exons, usually from* EWS*, which combine to produce a different-sized fusion transcript detectable by RT-PCR [21, 30]. Although the most common fusion occurs between* EWS* exon 7 and WT1 exon 8, designated 7/8, multiple combination variation transcripts have been reported [15, 30]. Splicing within* WT1* exon 9 to either insert or remove three amino acids produces additional transcripts [15, 30, 31]. If lysine, threonine, and serine are added, the isoform is designated as* EWS-WT1* (+KTS). If the amino acids are absent, the isoform is designated as* EWS-WT1* (−KTS) [30, 31]. Early work by Kim et al. suggested that the* EWS-WT1* (−KTS) isoform differentially experiences a gain of function mutation and transforms the fusion product into an oncogene that is not seen with* EWS-WT1* (+KTS) isoforms [30, 31]. However, in 2013, it was shown using murine embryonic fibroblasts that both isoforms could act as oncogenes if there was concurrent loss of p53 function [32]. Loss of p53 is also seen in retinoblastoma and sarcomas and was lost in our patient's tumor as part of the loss of 17p13.3-11.2 noted on a-CGH [32].

In addition to transcript length variations, two DSRCT cases were examined with FISH by La Starza et al. to reveal multiple copies of the* EWS-WT1* or* WT1-EWS* fusion products in patients with multiple copies of derivative chromosomes 11 or 22 [27]. DSRCTs with complex karyotypes, including numerical changes to chromosomes 1, 5, and 18, have also been reported [27]. Although the majority of our case's abnormalities were numerous base pair losses, there was also a loss of chromosome 18 (Table 1) that has been seen in DSRCTs [27].

Regardless of the translocation variant, the presence of the* EWS-WT1* fusion product by FISH or RT-PCR cements the diagnosis of a DSRCT, but there have been cases not showing this characteristic translocation. The appropriate diagnosis of these cases relies on a combination of clinical presentation, morphology, and immunohistochemistry to make the diagnosis. Using RT-PCR, de Alava et al. detected the fusion transcript in only 11/12 DSRCTs [33]. Other studies confirmed the fusion product by FISH or RT-PCR in 29/30 tumors (97%), while another study found the* EWS-WT1* fusion in 96% of 109 cases [18]. According to Lae et al., negative RT-PCR results could result from nonrepresentative tumor sampling or lack of viable RNA in the sample [18]. In addition to these technical reasons, the possibility of a DSRCT simply lacking the translocation must be considered. One of the first three cases of DSRCT with cytogenetic analysis was almost tetraploid, had multiple abnormalities, and lacked the characteristic translocation. One cell each in that case did show a del(22)(q12) and del(11)(p13) [19, 20]. When present, the translocation simplifies the diagnosis, but several examples of translocation-negative DSRCTs do exist.

Numerous cases of mesothelioma have also been subjected to karyotyping and a-CGH. Karyotyping revealed multiple chromosomal numerical changes with more losses than gains [34]. Recurrent changes identified by karyotype included monosomy of chromosomes 4 and 22, polysomy of chromosomes 5, 7, and 20, and losses of 1p21-p22, 3p21, 6q15-q21, 9p21-p22, 11p11-p13, 13q, 14q, and 22q12 [34]. Tumors subjected to a-CGH revealed the most frequent losses to be 1p11-p22, 3p21, 4q31.1-qter, 6q14, 6q22, 6q24, 6q25-qter, 8p12-p21, 8p21-qter, 9p21, 13q12-q14, 14q24-qter, 15q11.1-q21, and 22q13 [34]. Of these gene regions, 9p21-containing tumor suppressor gene* CDKN2A/p16INK4A*, is homozygously deleted in 100% of the mesothelioma cell lines tested by Klorin et al. [34]. This region, along with 22q12 containing the* NF2 gene*, which is inactivated in mesotheliomas, was also lost in our patient's tumor. Although the exact significance on histopathology or genetics of these losses is unknown, it is interesting to note that several of our patient's tumor abnormalities on Table 2 share overlapping losses with those described in malignant mesotheliomas [34].

Because DSRCTs generally express WT1 and are commonly located on peritoneal surfaces like mesotheliomas, authors have attempted to define DSRCTs as “a blastematous tumor derived of primitive mesothelium” [35]. The loss of 22q12.1-q13.33, along with several other shared losses found using a-CGH, seems to support a link between the primitive-appearing DSRCTs and mesotheliomas. Additional microarray data from DSRCTs are needed to determine which, if any, of our patient's chromosomal changes are recurrent to that tumor.

Our patient's tumor histologically appeared to have both mesotheliomatous and more primitive looking areas suggestive of desmoplastic small round cell tumor. The biphasic nature was confirmed with immunostaining, the mesothelial area marked with calretinin, WT1, D2-40, CK7, and CK5/6, while the small cell areas were positive for Golgi MyoD1, CD99, CD200, FLI1, and WT1. Interestingly, the cytologic features were characteristic of an epithelial mesothelioma rather than a small cell carcinoma or DSRCT. Although we hoped to confirm the diagnosis of a DSRCT component with FISH and later RT-PCR for the fusion product EWS-WT1, we conclude that the present case represents a small cell mesothelioma with the small cell component focally resembling a DSRCT and in other areas a blastemal-like mesothelioma.

## Figures and Tables

**Table 1:** Immunostaining results for large and small cell components of biphasic tumor.

Immunostain	Mesothelial areas	Small blue cell areas

MyoD1	Positive, diffuse	Positive, punctate Golgi
Manufacturer: Dako		
Clone: 5-8A		

CD99	Positive, diffuse	Positive, punctate Golgi
Manufacturer: Dako		
Clone: MIC2		

Myogenin	Negative	Negative
Manufacturer: Dako		
Clone: FSD		

CK7	Positive	Predominantly negative, few focal punctate positive cells
Manufacturer: Dako		
Clone: OV-TL-12/30		

CK20	Focal positive	Negative
Manufacturer: Dako		
Clone: Kg20-8		

TTF-1	Negative	Negative
Manufacturer: Leica		
Clone: SPT24		

CK5/6	Positive, membranous/cytoplasm	Negative
Manufacturer: Dako		
Clone: D5/16 B4		

Napsin	Negative	Negative
Manufacturer: Leica		
Clone: 1P64		

Synaptophysin	Negative	Negative
Manufacturer: Dako		
Clone: DAK-Synap		

Chromogranin	Negative	Negative
Manufacturer: Dako		
Clone: DAK-A3		

LCA (CD45)	Negative	Negative
Manufacturer: Dako		
Clone: 2B11 P07/26		

D2-40	Positive	Negative
Manufacturer: Dako		

Calretinin	Positive	Negative
Manufacturer: Dako		
Clone: Dako-Calret1		

CK (cocktail)	Positive, membranous	Positive, punctate
Manufacturer: Dako and Life technologies		
Clone: AE1/AE3 and Mak-6		

A103	Negative	Negative
Manufacturer: Dako		

S100	Negative	Focal positive
Manufacturer: Dako		

Desmin	Negative	Negative
Manufacturer: Dako		
Clone: D33		

BCL2	Negative	Weak positive
Manufacturer: Dako		
Clone: 124		

CD15	Negative	Positive, focal punctate
Manufacturer: Dako		
Clone: Carb3		

PLAP	Negative	Negative
Manufacturer: Dako		
Clone: 8A9		

HMB45	Negative	Negative
Manufacturer: Dako		

CD200	Positive	Positive
Manufacturer: R&D systems		

FLI1	Positive, patchy nuclear	Positive, nuclear
Manufacturer: BD Bioscience		
Clone: G146-222		

WT1	Positive, weak nuclear and focal cytoplasmic	Positive, weak nuclear and focal cytoplasmic
Manufacturer: Biocare Medical		
Clone Number: 6F-H2		

**Table 2:** Pleural tumor chromosomal alterations, cytogenetic band locations, genetic sizes, and the genes involved characterized by a-CGH.

Gain/loss	Chr.	Band	Genomic coordinates	Size (Mb)	# genes involved
Loss	1	p36.32-p36.13	chr1: 4762046–19269194	14.51	176
Loss	1	p31.3-p21.1	chr1: 54011592–105928237	41.92	207
Loss	3	p22.1-p11.1	chr3: 41537414–89032174	47.49	333
Loss	5	p15.2	chr5: 10871380–12541595	1.67	1
Loss	6	q16.1-q27	chr6: 95918819–170896238	74.98	378
Loss	9	p22.1-p21.2	chr9: 19296853–26822772	7.53	36
Loss	9	q33.2-q34.3	chr9: 124754535–141048319	16.29	309
Loss	10	p13-p12.31	chr10: 14966878–22520396	7.55	39
Loss	10	q23.1-q23.2	chr10: 82824485–89272483	6.45	26
Loss	10	q23.33-q25.1	chr10: 96270114–108891603	12.62	153
Loss	10	q25.3-q26.11	chr10: 118281211–119246215	0.97	12
Loss	10	q26.2-q26.3	chr10: 127801222–135425200	7.62	48
Loss	11	p15.5-p11.12	chr11: 205172–50406383	50.2	482
Loss	11	q11-q13.1	chr11: 55119736–64516115	9.4	260
Loss	13	q21.2-q31.3	chr13: 62076573–90289382	28.21	35
Loss	17	p13.3-p11.2	chr17: 51885–20317045	20.27	369
Loss	17	q11.2	chr17: 27804400–30770711	2.97	44
Loss	18	p11.32-p11.21	chr18: 118760–15083488	14.96	78
Loss	18	q11.1-q23	chr18: 18526965–78010032	59.48	227
Loss	20	q12-q13.13	chr20: 37642287–48165701	10.52	109
Loss	20	q13.33	chr20: 58988271–60137888	1.15	1
Loss	22	q12.1-q13.33	chr22: 26304781–51224252	24.92	347
Del	Y	q11.221-q12	chrY: 18548030–58909664	40.36	56
